# Inhibition of the mitochondria-shaping protein Opa1 restores sensitivity to Gefitinib in a lung adenocarcinomaresistant cell line

**DOI:** 10.1038/s41419-023-05768-2

**Published:** 2023-04-05

**Authors:** Masafumi Noguchi, Susumu Kohno, Anna Pellattiero, Yukino Machida, Keitaro Shibata, Norihito Shintani, Takashi Kohno, Noriko Gotoh, Chiaki Takahashi, Atsushi Hirao, Luca Scorrano, Atsuko Kasahara

**Affiliations:** 1 Department of Biology, University of Padua, 35121 Padua, Italy; 2 Veneto Institute of Molecular Medicine, 35129 Padua, Italy; 3 Laboratory of Pharmacology, School of Pharmaceutical Sciences, Wakayama Medical University, 25-1 Shichibancho, 640-8156 Wakayama, Japan; 4 Cancer Research Institute, Kanazawa University, 920-1192 Kanazawa, Japan; 5 Department of Veterinary Pathology, Nippon Veterinary and Life Science University Musashino, Tokyo, 180-8602 Japan; 6 Laboratory of Molecular Neuropharmacology, Graduate School of Pharmaceutical Sciences, Osaka University, 1-6 Yamadaoka, Suita, 565-0871 Osaka, Japan; 7 National Cancer Center Research Institute, 104-0045 Tokyo, Japan; 8 Institute for Frontier Science Initiative, Kanazawa University, 920-1192 Kanazawa, Japan; 9 WPI Nano Life Science Institute (WPI- Nano LSI), Kanazawa University, 920-1192 Kanazawa, Japan

**Keywords:** Apoptosis, Non-small-cell lung cancer, Cancer therapeutic resistance, Energy metabolism

## Abstract

Drug resistance limits the efficacy of chemotherapy and targeted cancer treatments, calling for the identification of druggable targets to overcome it. Here we show that the mitochondria-shaping protein Opa1 participates in resistance against the tyrosine kinase inhibitor gefitinib in a lung adenocarcinoma cell line. Respiratory profiling revealed that oxidative metabolism was increased in this gefitinib-resistant lung cancer cell line. Accordingly, resistant cells depended on mitochondrial ATP generation, and their mitochondria were elongated with narrower cristae. In the resistant cells, levels of Opa1 were increased and its genetic or pharmacological inhibition reverted the mitochondrial morphology changes and sensitized them to gefitinib-induced cytochrome *c* release and apoptosis. In vivo, the size of gefitinib-resistant lung orthotopic tumors was reduced when gefitinib was combined with the specific Opa1 inhibitor MYLS22. The combo gefitinib-MYLS22 treatment increased tumor apoptosis and reduced its proliferation. Thus, the mitochondrial protein Opa1 participates in gefitinib resistance and can be targeted to overcome it.

## Introduction

Lung cancer is one of the most fatal cancers worldwide. More than 85% of lung cancer cases are classified as non-small-cell lung cancer (NSCLC), which can be histologically subdivided into adenocarcinoma, squamous cell carcinoma, and large cell carcinoma. In addition to surgical removal of early-stage NSCLC, chemotherapy had been the only available tool for advanced tumor until targeted drugs that act on molecularly defined NSCLC liabilities have been discovered. For example, for NSCLC adenocarcinoma with gain of function mutations in Epidermal Growth Factor Receptor (EGFR) as well as with *EGFR* gene amplification, tyrosine kinase inhibitors (EGFR-TKIs) have been developed [1]. EGFR-TKIs act well as first-line of treatment for patients with *EGFR* mutations [2, 3]. However, the appearance of lung adenocarcinoma cells resistant to the treatment leads to relapse with scant therapeutic options. Several molecular mechanisms underlie this acquired resistance to EGFR-TKIs. They include the emergence of novel on-target mutations that render cancer insensitive to the TKI, as well as off-target mechanisms that involve pathways including HER2, HGF/c-MET, VEGF IGF1, EMT, and STAT3, PTEN, RAS, and BRAF [4, 5]. Often, resistance develops in a subset of tumor cells with stem-like properties. These cancer stem-like cells (CSCs) are ultimately responsible for recurrence and drug resistance. A fraction of these CSCs also exhibits the ability to regrow after years, causing recurrence. Thus, tumor eradication would require targeted therapies against CSCs.

Interestingly, CSC metabolism in cholangiocarcinoma [6], pancreatic adenocarcinoma [7], and glioma [8] seems to differ from that of the bulk tumor, displaying oxidative features instead of the glycolytic signature of the cancer parenchyma. Whether this occurs also in NSCLC is unclear. Altogether, mitochondrial oxidative metabolism might be a key factor for resistance to targeted therapies and an appealing target to eradicate CSCs and recurring tumors.

In addition to their role in oxidative metabolism and ATP production through oxidative phosphorylation, mitochondria are an appealing target to circumvent targeted therapy resistance also because of their crucial roles in metabolic pathways, calcium and redox homeostasis, and apoptosis [9–12]. These multiple mitochondrial functions are reflected by their extremely dynamic morphology, which results from their fusion and division [13] controlled by core mitochondria-shaping proteins. The dynamin-related GTPases Mitofusin (Mfn) 1 and 2, and Optic Atrophy 1 (Opa1) fuse mitochondria, and the cytosolic dynamin-related protein 1 (Drp1) divides mitochondria [11]. Opa1 is not only essential for mitochondrial inner membrane fusion, but it is also a key player in apoptosis, where it protects cells by keeping the cristae junction tight to prevent complete cytochrome *c* release [14, 15]. Moreover, Opa1 is required for the stabilization of the respiratory chain supercomplexes and for efficient mitochondrial oxidative metabolism [16]. Consequently, carbon flux along the tricarboxylic acid cycle (TCA) is increased in Opa1 overexpressing cells [17].

The pro-fusion mitochondria-shaping proteins also participate in establishing other features of cancer cells. For example, Opa1 is required for cancer angiogenesis [18], is upregulated in Cisplatin resistant lung adenocarcinomas [19], in Venetoclax-resistant acute myeloid leukemia cells [20], and in triple-negative breast cancer (TNBC), the growth of which is curtailed by genetic and pharmacological Opa1 inhibition [21]. In lung adenocarcinoma patient samples also *MFN2* is overexpressed, and its downregulation in a lung adenocarcinoma cell line decreases proliferation and invasion [22]. In addition, mitochondria appear elongated upon acute treatment of lung adenocarcinoma cells with gefitinib [23]. Nevertheless, whether mitochondria-shaping proteins participate in resistance to gefitinib and can be targeted to revert it is unknown. We, therefore, set out to investigate the role of mitochondria and mitochondria-shaping proteins in gefitinib-resistant lung adenocarcinoma. We show that levels of Opa1 are increased in a well-characterized gefitinib-resistant adenocarcinoma cell line and that its genetic or pharmacological inhibition reverts gefitinib resistance in vitro and in vivo. Our data nominate Opa1 as a target to overcome gefitinib resistance in NSCLC.

## Results

### Gefitinib-resistant lung adenocarcinoma cells rely on mitochondrial ATP production

To investigate the role of mitochondria in resistance to gefitinib, we capitalized on PC9M2, a gefitinib-resistant lung adenocarcinoma cell line that was generated by long-term exposure of a sensitive PC9 cell line carrying a 5 amino acid deletion in the EGFR tyrosine kinase domain targeted by gefitinib [5]. PC9M2 cells do not display any additional EGFR mutations but show increased Akt-β-catenin signaling [5].

**Fig. 1:**
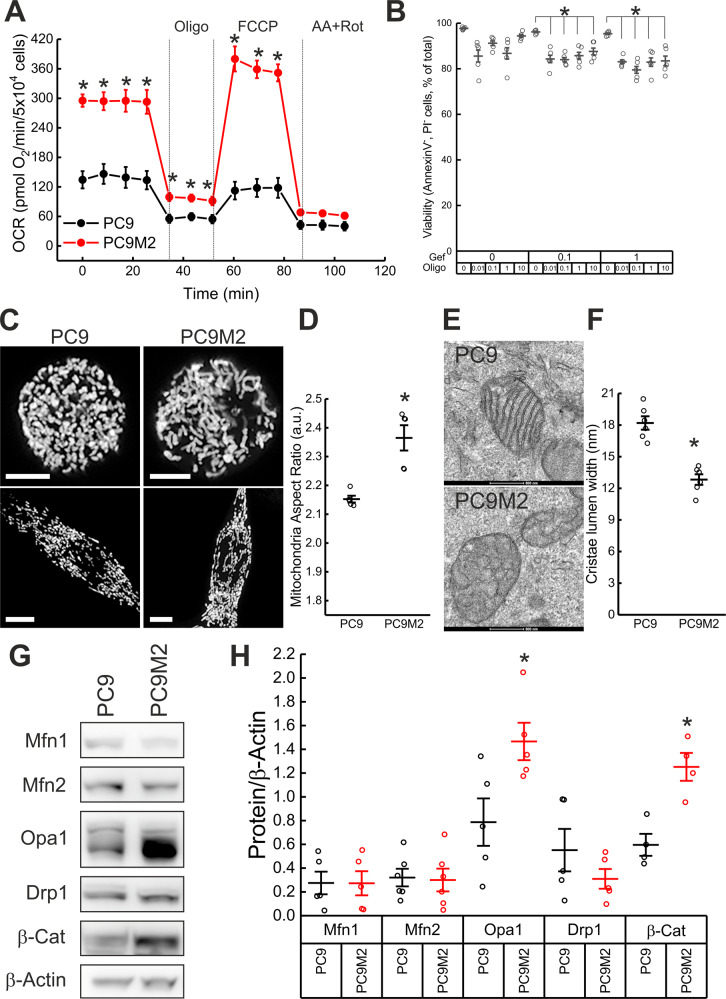
Increased Opa1 levels, mitochondrial elongation, and narrow cristae in Gefitinib-resistant PC9M2 cells. **A** Oxygen consumption rates were measured by Seahorse analyzer. Where indicated, 0.75 µM Oligomycin (Oligo), 1 µM FCCP, 1 µM Antimycin A (AA) and 1 µM Rotenone (Rot) were injected. Data represent mean ± SEM of three independent experiments. *p* values were calculated using a two-sided Student’s *t* test (*p < 0.05). **B** PC9M2 cells were treated with the indicated concentrations of the indicated compounds and viability was assessed by Annexin-V/PI staining. Data represent mean ± SEM of five independent experiments. *p* values were calculated using a Tukey test (*p < 0.05). **C** Representative maximum projections of confocal Z-stacks of mtYFP fluorescence in PC9 and PC9M2 cells transfected with mtYFP. Twenty-four hours after transfections, confocal Z-stacks were acquired. Bar 10 μm. **D** Morphometric analysis of mitochondrial aspect ratio in PC9 and PC9M2 cells in experiments as in **C**. Data represents mean ± SEM of five independent experiments. *p* values were calculated using a non-parametric Kolmogorov–Smirnov Test (*p < 0.05). **E** Representative electron micrographs of PC9 and PC9M2 cells. Bar 0.5 μm. **F** Quantification of cristae lumen width in six independent experiments as in **E** (*n* = 100–150 mitochondria per condition, 3–4 cristae per mitochondria). p values were calculated using a non-parametric Kolmogorov–Smirnov Test (*p < 0.05). **G** PC9 and PC9M2 cells were lysed, and equal amounts of proteins (30 µg) were separated by SDS-PAGE and immunoblotted using the indicated antibodies. **H** Mean ± SEM of densitometric data from five independent experiments as in **G**. *p* values were calculated using a non-parametric Scheffe test (*p < 0.05).

First, we compared metabolism in PC9 and PC9M2 cells by Seahorse flux analysis. Oxygen consumption rate (OCR) was twofold higher in the gefitinib-resistant cells (Fig. 1A). Mitochondrial ATP production sustained resistance of PC9M2 cells to Gefitinib, as indicated by the loss of viability when these cells were treated with gefitinib and the mitochondrial ATP synthase inhibitor oligomycin (Fig. 1B). This occurred despite the finding that extracellular acidification rate (ECAR), a proxy of glycolytic metabolism that can in principle supply ATP and intermediates for cell growth, was increased in PC9M2 cells (Supplementary Fig. 1). Thus, PC9M2 cells display an oxidative metabolism, and inhibition of mitochondrial ATP production by oligomycin sensitizes them to gefitinib.

We next addressed whether the observed oxidative metabolism phenotype was accompanied by mitochondrial morphological changes. Confocal imaging of a transfected mitochondrially targeted yellow fluorescent protein (mtYFP) indicated that mitochondria were longer in PC9M2 than in PC9 cells, irrespective of the overall morphology of the cells in the culture dish (Fig. 1C, D). Transmission electron microscopy confirmed this mitochondrial elongation and revealed that in PC9M2 cells cristae lumen was narrower (Fig. 1E, F). Altogether, these experiments indicate that gefitinib-resistant lung adenocarcinoma PC9M2 cells rely on oxidative metabolism and display elongated mitochondria with narrow cristae.

### Opa1 sustains the mitochondrial phenotype in Gefitinib-resistant PC9M2 cells

To understand the molecular basis for the mitochondrial phenotype observed in PC9M2 cells, we first measured whether components of the mitochondrial respiratory chain were more abundant. Immunoblotting revealed no changes in levels of complex I or complex II components of the respiratory chain between PC9M2 and PC9 cells (Supplementary Fig. 2A, B). We, therefore, turned our attention to mitochondria-shaping proteins that might account for the observed functional and morphological changes. While levels of Mfn1, Mfn2, and Drp1 were not changed, Opa1 expression was increased in PC9M2 compared to PC9 cells (Fig. 1G, H). Opa1 is proteolytically cleaved by the inner membrane-AAA (i-AAA) protease Yme1 [24], and by stress-activated zinc metallopeptidase OMA1 [24, 25] to produce short-Opa1 (s-Opa1) from long-Opa1 (l-Opa1). Levels of YME1L and of OMA1 were, however, unchanged in PC9M2 cells (Supplementary Fig. 2A, B). Thus, in PC9M2 cells total mitochondrial content is not changed, but Opa1 is specifically upregulated, a feature that is shared with several other conditions of cancer cell resistance to chemotherapy and targeted therapeutics [19, 20].

**Fig. 2:**
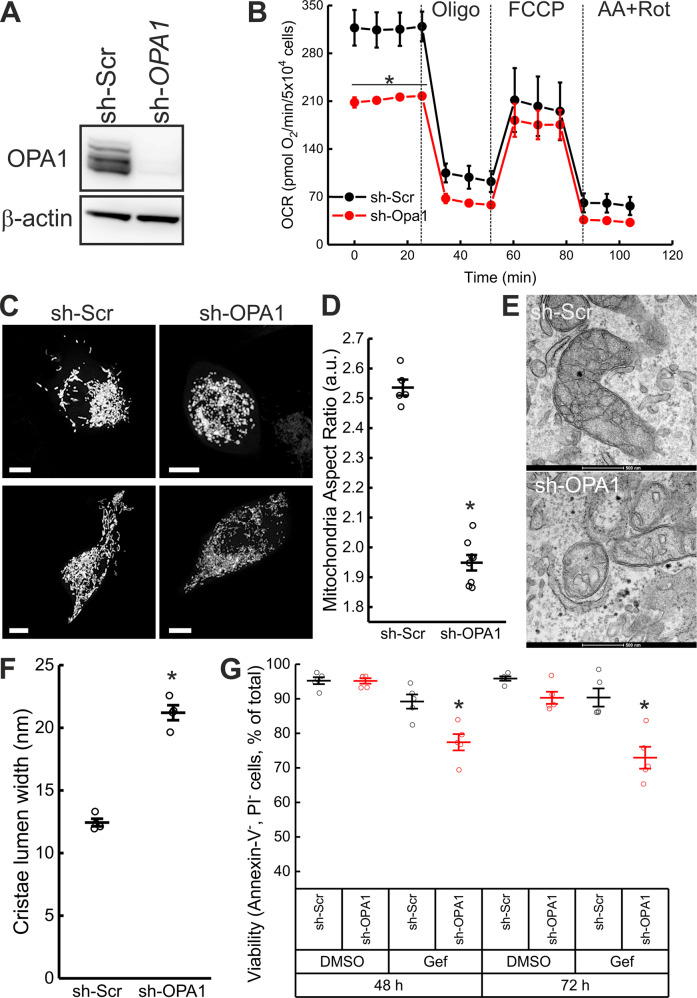
Opa1 sustains PC9M2 cells resistance to gefitinib. **A** PC9M2 cells infected with sh-Scramble (sh-Scr) and sh-*Opa1* lentiviruses were lysed, and equal amounts of proteins (30 µg) were separated by SDS-PAGE and immunoblotted using the indicated antibodies. **B** Oxygen consumption rates were measured by Seahorse analyzer. Where indicated, 0.75 µM Oligo, 1 µM FCCP, 1 µM AA, and 1 µM Rot were injected. Data represent mean ± SEM of three independent experiments. *p* values were calculated using a two-sided Student’s *t* test (*p < 0.05). **C** Representative maximum projections of confocal Z-stacks of mtYFP fluorescence in PC9M2 cells transfected with mtYFP and infected with the indicated lentiviruses. Bar 10 μm. **D** Morphometric analysis of mitochondrial aspect ratio in sh-Scr and sh-*Opa1* cells in experiments as in **C**. Data represent mean ± SEM of three independent experiments The *p* values were calculated using a non-parametric Kolmogorov–Smirnov Test (*p < 0.05). **E** Representative electron micrographs of sh-Scr and sh-*Opa1* cells. Cells were fixed, and TEM images of randomly selected fields were acquired. Bar 0.5 μm. **F** Quantification of cristae lumen width in **E** from independent experiments (*n* = 100–150 mitochondria per condition, 3–4 cristae per mitochondria). p values were calculated using a non-parametric Kolmogorov–Smirnov Test (*p < 0.05). **G** sh-Scr and sh-*Opa1* cells were treated with DMSO or 1 µM gefitinib (Gef) for the indicated time and viability was assessed by Annexin-V/PI staining. Data represent mean ± SEM of five independent experiments. *p* values were calculated using a non-parametric Scheffe test (*p < 0.05).

To understand whether these observed changes were caused by the increased Opa1 levels, we efficiently downregulated *OPA1* in PC9M2 cells using short hairpin RNAs (Fig. 2A). *OPA1* downregulation resulted in decreased basal OCR, whereas FCCP-stimulated OCR was not affected, probably because of FCCP toxicity after shRNA viral delivery (Fig. 2B). As expected, *OPA1* downregulation resulted in the appearance of shorter mitochondria (Fig. 2C, D) with a wider cristae lumen (Fig. 2E, F). In conclusion, in gefitinib-resistant PC9M2 cells, Opa1 upregulation is responsible for the observed mitochondrial changes.

### Opa1 expression levels are associated with worse prognosis of lung adenocarcinoma patients undergoing chemotherapy

**Fig. 3:**
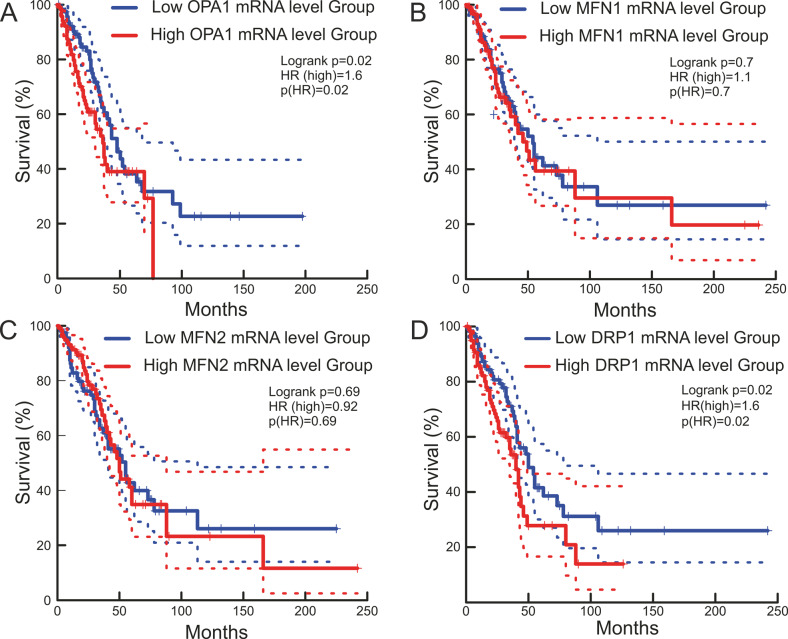
Lung adenocarcinoma patients with high *OPA1* and *DRP1* mRNA levels treated with chemotherapy display a worse prognosis. Kaplan–Meier survival curves of lung adenocarcinoma patients in PanCancer Atlas. Red and blue lines represent survival curves of patients with lung tumor tissues with high and low expression levels of *OPA1* (**A**) *MFN1* (**B**), *MFN2* (**C**), and *DRP1* (**D**) mRNA. The cutoff value was determined as the quantile value of the expression levels of each mRNA (*n* = 120). Dashed lines indicate 95% confidence intervals. The *p* values were calculated using a log-rank test. The hazard ratio was calculated with Cox PH Model. *HR* hazard ratio.

To understand whether the observed Opa1 upregulation in these gefitinib-resistant lung adenocarcinoma cells was indicative of a clinically relevant condition, we investigated publicly available databases and bioinformatically evaluated whether a “mitochondria-shaping proteins” signature existed in chemoresistant lung adenocarcinoma (LUAD) patients. To this end, in the PanCancer Atlas database we stratified patients who received chemotherapy based on mRNA expression groups of mitochondria-shaping proteins and analyzed overall censored survival in high and low mRNA groups. Prognosis was not different in *MFN1*, or *MFN2* high and low groups, whereas it was significantly worse for the high *OPA1* patients’ group (Fig. 3A–C). We also observed a similarly worse prognosis for the high *DRP1* patients’ group (Fig. 3D) These analyses indicate that higher levels of the mitochondrial fusion gene *OPA1*, but not of the other fusion genes *MFN1* and *MFN2*, are associated with worse prognosis in LUAD patients undergoing therapy. Similarly, high *DRP1* levels are associated with worse prognosis. However, deletion of Drp1 does not affect growth of K-Ras mutated LUAD [26]. Thus, high Opa1 LUAD might be less responsive to therapy and/or prone to relapse because of OPA1 overexpression, calling for an analysis of the role of this mitochondria-shaping protein in LUAD resistance to therapy.

### Opa1 sustains PC9M2 cells resistance to gefitinib

Because our cell biology and bioinformatic data suggested a role for Opa1 in the phenotype of the gefitinib-resistant PC9M2 cells as well as in defining prognosis of chemotherapy-treated LUAD patients, we decided to investigate whether genetic and pharmacological inhibition of Opa1 could restore sensitivity to gefitinib.

**Fig. 4:**
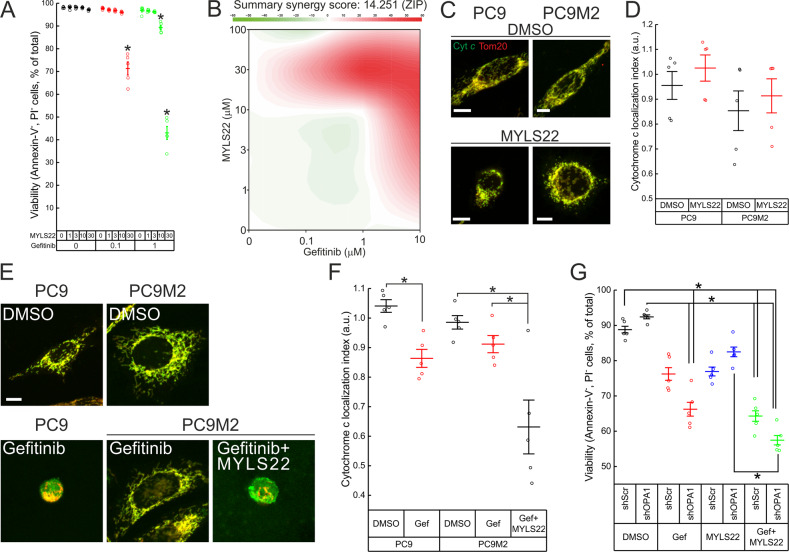
MYLS22 synergizes with gefitinib to induce Opa1-dependent mitochondrial apoptosis in PC9M2 cells. **A** PC9M2 cells were treated with the indicated concentrations of the indicated compounds and viability was assessed by Annexin-V/PI staining. Data represent mean ± SEM of five independent experiments. p values were calculated using a non-parametric Scheffe test (*p < 0.05). **B** Combination index was assessed by exposing PC9M2 cells to varied MYLS22 and Gef concentration combinations, which resulted in a significant synergistic effect. Synergy scores based on the zero-potency interaction model are visualized as 2D landscape. The overall levels of synergy effects of the two compounds are shown as delta scores. **C** Representative confocal images of subcellular cytochrome *c* distribution. PC9 and PC9M2 cells were untreated or treated for 72 h with 30 µM MYLS22, fixed and immunostained for cytochrome *c* (green) and Tom20 (red). Bar, 10 µm. **D** Localization index of cytochrome c was calculated from at least 83 images. Data represent mean ± SEM of five independent experiments. **E** Representative confocal images of subcellular cytochrome *c* distribution. PC9 and PC9M2 cells were treated with DMSO, 1 µM Gef, 30 µM MYLS22 and 1 µM Gef+30 µM MYLS22 for 72 h, fixed and immunostained in experiments as in **C**. Bar, 10 µm. **F** Localization index of cytochrome *c* was calculated from at least 31 images. Data represent mean ± SEM of five independent experiments. *p* values were calculated using a paired sample signed test between DMSO and gefitinib treated PC9 cells and using a non-parametric Scheffe test among DMSO, gefitinib and gefitinib+ MYLS22 treated PC9M2 cells. **G** PC9M2-sh-Scr and sh-Opa1 cells were treated with DMSO, 1 µM Gef, 30 µM MYLS22, and 1 µM Gef+30 µM MYLS22 and viability was assessed by Annexin-V/PI staining. Data represent mean ± SEM of five independent experiments. The p values were calculated using a non-parametric Scheffe test (*p < 0.05).

First, we tested if gefitinib could kill PC9M2 cells where we had downregulated Opa1 expression by the delivery of the efficient shRNA that normalized respiration and morphology/ultrastructure. Indeed, *OPA1* downregulation could restore the sensitivity of these cells to gefitinib, without being per se toxic (Fig. 2G). Next, we turned to MYLS22, a specific Opa1 inhibitor recently developed in our lab that curtails Opa1-dependent tumor angiogenesis and growth [18] and is efficacious against TNBC cells in vitro and in vivo [21]. We, therefore, tested whether MYLS22 recapitulated the effects of *OPA1* silencing in PC9M2 cells. While administration of MYLS22 was not toxic in PC9M2 cells, it restored sensitivity to gefitinib (Fig. 4A). Conversely, MYLS22 did not significantly increase cell death of PC9 cells exposed to gefitinib, further indicating that the sensitization effect of MYLS22 on PC9M2 cells relies on the observed Opa1 overexpression in this resistant cell line (Supplementary Fig. 3). We addressed if the observed effect in PC9M2 cells was merely additive or if it reflected true synergism between Opa1 inhibition and gefitinib. To this end, we performed a synergism analysis in a Zero interaction potency (ZIP) model that computes the drug interaction relationship by comparing the change in the potency of the dose–response curves between individual drugs and their combinations [27]. SynergyFinder (https://synergyfinder.fimm.fi/) computed an average synergism of 14.25 in the ZIP model, with the most synergistic area around 30 µM MYLS22 and 1 µM gefitinib (Fig. 4B).

Comforted by these results, we comprehensively analyzed the effects of MYLS22 on PC9M2 cells. MYLS22 recapitulated the mitochondrial fragmentation (Supplementary Fig. 3A, B) and the reduction of OCR (Supplementary Fig. 3C) observed when we downregulated *OPA1* in PC9M2 cells. Mechanistically, we ascribed the restoration of gefitinib sensitivity to the ability of MYLS22 to increase cytochrome *c* release upon gefitinib treatment. Gefitinib was indeed very inefficient in inducing cytochrome c release in PC9M2 cells and MYLS22 did not induce cytochrome *c* release from mitochondria per se in both PC9 and PC9M2 cells. This result was expected, given that cytochrome *c* release requires mitochondrial outer membrane permeabilization, and activation of the Opa1-controlled cristae remodeling pathway is per se not sufficient to elicit cytochrome *c* release [28] (Fig. 4C–F). Conversely, the combination of gefitinib and MYLS22 resulted in complete cytochrome *c* release from mitochondria (Fig. 4E, F). Finally, we tested whether MYLS22 required Opa1 to restore sensitivity to gefitinib. In PC9M2 cells, apoptosis induced by gefitinib was increased to comparable levels by *OPA1* silencing or treatment with MYLS22 in cells infected with a control shRNA. In PC9M2 cells where we silenced OPA1, MYLS22 did not display any additive effect on the stimulation of apoptosis by gefitinib (Fig. 4G). Altogether, these experiments establish that MYLS22 restores gefitinib-induced mitochondrial apoptosis in PC9M2 cells.

### MYLS22 restores gefitinib sensitivity in an in vivo PC9M2 xenograft model

**Fig. 5:**
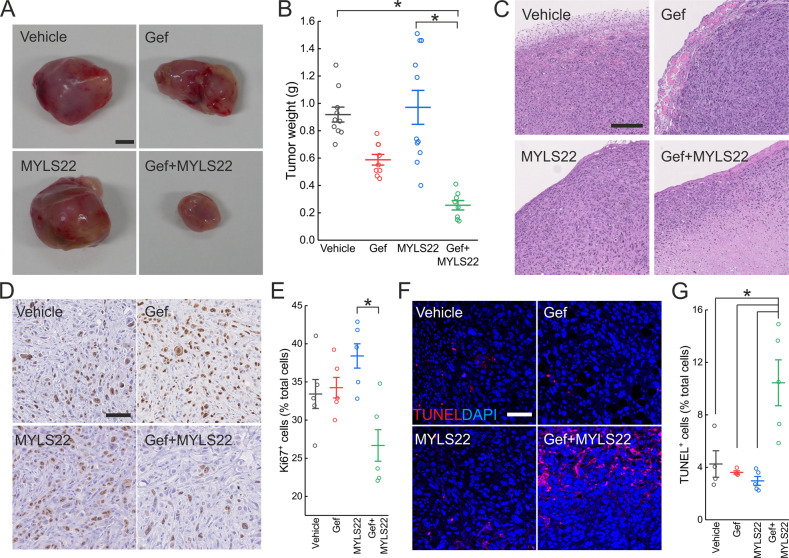
MYLS22 restores gefitinib sensitivity in an in vivo PC9M2 xenograft model. **A** Representative images of PC9M2 xenografts. 1 × 10^6^ PC9M2 cells were inoculated into 6 weeks old KSN/Slc male mice. After ~2 weeks, when tumor size approached 150 mm^3^, tumor-burdened mice were treated as indicated every 2 days, and after 2 further weeks mice were euthanized and tumors were explanted for analysis. Scale bar, 0.5 cm. **B** Quantification of tumor weight from experiments as in **A**. Data represent mean ± SEM of *n* ≥ 8 mice/group. *p* values were calculated using a Dunn’s test among vehicle, OPA1i, gefitinib, and gefitinib+OPA1i treated mice (*p < 0.05). **C** Representative images of HE-stained, paraffin-embedded tumor sections from experiments as in **A**. Scale bar, 250 µm. **D** Representative images of Ki67-immunohistochemical staining of xenograft tumors from experiments as in **A**. Scale bar, 70 µm. **E** Quantification of Ki67-positive cells from experiments as in **D**. Ki67-positive cells in five ~0.5 mm^2^ areas sampled from a periphery of each tumor were counted. Data represent mean ± SEM of five xenografts/group. *p* values were calculated using a Tukey’s test (*p < 0.05). **F** Representative images of TUNEL-immunohistochemical staining of xenograft tumors from experiments as in **A**. Scale bar, 100 µm. **G** Quantification of TUNEL-positive cells from experiments as in **F**. TUNEL-positive cells in at least eight ~0.5 mm^2^ areas sampled from each tumor were counted. Data represent mean ± SEM of 4–5 xenografts/group. *p* values were calculated using a non-parametric Scheffe test (*p < 0.05). Statistical outliers were identified using Grubb’s test and removed from all measures.

We next tested whether MYLS22 synergized with gefitinib in vivo. To this end, we implanted PC9M2 xenografts in adult mice and after two weeks, when the tumor was already clinically palpable, we treated mice with gefitinib alone or in combination with MYLS22. While as expected these xenografts were totally insensitive to gefitinib, two weeks of treatment with the gefitinib-MYLS22 combo reduced the weight of the PC9M2 tumors by >70% (Fig. 5A, B). We explanted these tumors and characterized them by histology. In hematoxylin-eosin-stained sections of tumors treated with the gefitinib-MYLS22 combo, we retrieved a large central area devoid of visible cells, suggestive of massive cell death induction (Fig. 5C). We, therefore, analyzed the effects of the gefitinib-MYLS22 combo in the pericentral region of the treated tumors. Here, we found that the gefitinib-MYLS22 combo reduced proliferation, as indicated by immunohistochemistry analysis of the Ki67 proliferation marker (Fig. 5D, E), and increased apoptosis, as indicated by the increased frequency of TUNEL-positive cells (Fig. 5F, G). Collectively, these data indicate that the MYLS22 reverts gefitinib resistance of lung adenocarcinoma cells also in vivo, restraining tumor proliferation and inducing tumor cell death.

## Discussion

Mitochondria are central executioners of cell death and are often involved in the processes of resistance to classic and targeted anticancer drugs, including gefitinib in lung adenocarcinomas. Our studies nominate the mitochondria-shaping protein Opa1 as a promising target to overcome gefitinib resistance in lung adenocarcinoma. Chronic gefitinib treatment renders lung adenocarcinoma cells exquisitely oxidative, with elongated mitochondria displaying narrow cristae lumen. These mitochondrial changes are due to increased Opa1 levels. Genetic as well as pharmacological Opa1 inhibition not only corrects them, but more importantly reverts gefitinib resistance in vitro and in vivo.

Mitochondria are emerging as key organelles in the development of gefitinib resistance. Indeed, in line with our results, gefitinib-resistant lung cancer cell lines rely on oxidative metabolism that once targeted can restore sensitivity to gefitinib [29, 30]. The role of mitochondria in mediating gefitinib resistance is not limited to lung cancer but has been identified also for example in colorectal cancer, where the gefitinib resistance can be overcome by the mitochondriotoxic drug salinomycin [31, 32]. Interestingly, PC9M2 cells rely on mitochondrial 1-carbon metabolism to replenish their purine pool, and deletion of the 1-carbon metabolism mitochondrial enzyme methylenetetrahydrofolate dehydrogenase 2 (MTHFD2) restores gefitinib sensitivity, further pointing to the multifaceted role of mitochondria in gefitinib resistance [33]. Nevertheless, the role of mitochondrial dynamics in gefitinib resistance in lung adenocarcinoma was unclear.

Acute gefitinib treatment appears to elongate mitochondria in lung adenocarcinoma cells [34], suggesting gefitinib can impinge on the mitochondrial dynamics machinery. Our profile of core mitochondria fusion and fission proteins in PC9 vs. PC9M2 cells points to a unique signature of Opa1 upregulation. This is consistent with previous findings of Opa1 upregulation in cisplatin-resistant lung cancer cells [19]. Unfortunately, our attempts to identify the upstream regulators that alter the expression of Opa1 in this lung adenocarcinoma cell line were unsuccessful. Upon acute gefitinib treatment of PC9 cells the mRNA levels of *C*-*MYC* increased, but its ectopic expression in untreated PC9 cells did not change Opa1 levels. Similarly, expression of miR34a, which was significantly downregulated in PC9M2 cells and upon acute gefitinib treatment of PC9 cells, did not affect Opa1 levels. Therefore, multiple yet uncharacterized pathways may be involved in the upregulation of Opa1 in gefitinib-resistant lung adenocarcinoma cells.

We identified MYLS22 in screening for inhibitors of GTPase activity of recombinant Opa1 [35] performed to identify inhibitors of Opa1 pro-fusion and cristae sculpting activities that both require its GTPase activity [14, 36]. Not surprisingly, Opa1 inhibition using MYLS22, therefore, recapitulates the effects of genetic Opa1 deletion, in PC9M2 cells (here) as well as in endothelial cells and in TNBC cells, and orthotopically implanted tumors [18, 21]. MYLS22 has been validated independently as an Opa1 inhibitor that aggravates alveolar cell necroptosis and sensitizes T helper 17 cells to apoptosis [37, 38]. Mechanistically, our results corroborate the concept that Opa1 is a key molecule to regulating cytochrome *c* egress from mitochondria. Indeed, PC9M2 cells are remarkably resistant to cytochrome *c* release induced by gefitinib, one of the mitochondrial consequences caused by this EGFR-TKI [39]. However, when PC9M2 cells are treated with gefitinib in combination with MYLS22, cytochrome *c* is rapidly and fully released in the cytosol. These results are in line with the finding that cristae are narrower in PC9M2 cells, a hallmark of Opa1 overexpression in multiple healthy tissues and in cancer cells [20, 40]. Narrowing of cristae junctions upon Opa1 overexpression limits intramitochondrial cytochrome *c* redistribution and hence the amount of cytochrome *c* that can be released via selective outer mitochondrial membrane permeabilization pathways [14, 41]. This is of paramount importance in the context of cancer cells, where higher cytosolic levels of cytochrome *c* must be reached to activate effector caspases [42]. In sum, MYLS22 appears as a powerful pro-death compound that can be used alone or in combination with targeted therapeutics in conditions where Opa1 mechanistically participates in the emergence of chemoresistant cancer cells.

Resistance and/or relapse following chemotherapy or targeted therapy is a clinical challenge in the management of LUAD patients [43]. Our bioinformatic analysis reveals that the probability of survival is lower for patients with high *OPA1* expression, raising the possibility that Opa1 can be used in the clinics to stratify patients and more importantly can be specifically targeted in a subset of patients for whom therapeutic options are currently very scant.

## Materials and methods

### Cell culture

PC9 and PC9M2 lung adenocarcinoma cells [44] were cultured in RPMI-1640 medium (Wako) supplemented with 10% fetal bovine serum (FBS) (Gibco), 50 U/ml penicillin (Gibco) and 50 mg/ml streptomycin (Gibco).

### Molecular biology

mtYFP has been described previously [45]. Downregulation of *OPA1* in PC9M2 cells was achieved via RNA interference using a lentiviral-based small hairpin (sh) RNA. The target sequence CCGGACCTTAGTGAATATAAA (TRCN0000082846) was cloned into pLKO.1 following supplier’s instructions (Addgene).

### Transfection, lentivirus infection, and generation of stable clones

Transfection was performed using Lipofectamine 2000 (Invitrogen) following the manufacturer’s instructions. Cells were infected with lentiviruses using 10 µg/ml polybrene, and 24 h after infection cells were selected using 2 µg/ml puromycin.

### Immunoblotting

PC9 and PC9M2 cells were harvested and lysed in RIPA buffer (150 mM NaCl, 1% Nonidet P-40, 0.25% deoxycholate, 1 mM EDTA, 50 mM Tris, pH 7.4) in the presence of complete protease inhibitor mixture (Roche). The extracted proteins were separated using 4–12% Tris-MOPS gel (NuPAGE, Invitrogen) and transferred onto polyvinylidene difluoride membrane (PVDF, BioRad). The membranes were probed using the following antibodies: monoclonal anti-MFN1 (1:1000, Abcam, ab57602), monoclonal anti-MFN2 (1:1000, Abnova, H00009927-M03), monoclonal anti-Opa1 (1:1000, BD Biosciences, 612607), monoclonal anti-DLP1 (1:1000, BD Biosciences, 611112), monoclonal anti-β-catenin (1:1000, BD Biosciences, 610154), rabbit polyclonal anti-OMA1 (1:1000, Proteintech, 17116-1-AP), rabbit polyclonal anti-YME1L (1:1000, Proteintech, 11510-1-AP), monoclonal anti-NDUFA9 (1:1000, Abcam, ab14713), monoclonal anti-SDHA (1:1000, Abcam, ab14715), and monoclonal anti-β-actin (1:100000, Sigma, A5316). Isotype-matched, horseradish peroxidase-conjugated secondary antibodies (Amersham) were used, followed by detection using chemiluminescence (Amersham).

### Confocal imaging

For imaging of the mitochondrial network, 1 × 10^5^ PC9 or PC9M2 cells were seeded onto 24-mm-round glass coverslips and transfected with mtYFP. After 24 h, cells expressing mtYFP were excited using the 488 nm line of the Argon laser with an HC PL APO ×63/1.20 W CORR CS2 0/D objective (Leica TCS SP8). Morphometric analysis was performed using ImageJ (NIH) and MitoSegNet [46]. For confocal z-axis stacks of the mitochondrial network, stacks of 20 images separated by 0.34 µm along the *z* axis were acquired for steady-state 3D imaging. Maximum projections of confocal Z-stacks were performed using the appropriate plugin of ImageJ. The processed 2D images were binarized using MitoSegNet, segmented, and an ellipse was fitted to each segmented mitochondrion using ImageJ. The major and minor axis lengths of the fitted ellipse were measured, and the aspect ratio was calculated as the major axis length divided by the minor axis length. At least 1500 mitochondria per experiment were analyzed.

### Cytochrome c immunolocalization

For cytochrome *c* immunolocalization, cells were grown on coverslips coated with Cell-Tak adhesive (Corning) and treated with the indicated concentration of gefitinib, MYLS22, or DMSO in the presence of 50 µM zVAD-fmk (Merck Millipore). After 72 h, cells were fixed and immunostained, as described [47], with anti-cytochrome *c* antibody (1:200, BD Biosciences, 556432) and anti-TOM20 antibody (1:200, Santa Cruz Biotechnology, Sc-11415), and Alexa Fluor 488 goat anti-mouse IgG (1:200, Thermo Fisher Scientific, A-11029) and Alexa Fluor 568 donkey anti-rabbit IgG (1:200, Thermo Fisher Scientific, A-10042) as secondary antibodies. For cytochrome *c* and TOM20 detection, green and red channel images respectively were acquired simultaneously using two separate color channels on the detector assembly of a Zeiss LSM 700 confocal microscope. The localization index was calculated as described [48].

### Transmission electron microscopy

Cells were fixed for 30 min at room temperature using glutaraldehyde at a final concentration of 2.5% (v/v) in 0.1 M sodium cacodylate at pH 7.4. Embedding and staining were performed as described [45]. Thin sections were imaged on a Tecnai-G2 transmission electron microscope operating at 100 kV. Images were captured using a Veleta (Olympus Imaging System) digital camera (pixel size at ×46,000 magnification with screen magnification of ×3: 0.1 × 0.1 nm). Cristae lumen width was quantified using the ImageJ Freehand line selection tool.

### Cell viability assay

For apoptotic/necrotic cell death detection, 2 × 10^4^ PC9 and PC9M2 cells grown in 12-well plates were treated as indicated and stained with propidium iodide and annexin-V-Alexa488 (Roche) according to the manufacturer’s protocol. Apoptotic and necrotic cell death were detected using flow cytometry (FACSLyric).

### Analysis of mitochondrial respiration

Intact cellular respiration was analyzed using the Seahorse XF24 analyzer (Seahorse Bioscience). Respiration was measured under basal condition, and in the presence of 0.75 µM of the ATP synthase inhibitor oligomycin, 1 µM FCCP, an uncoupler, 1 µM rotenone, a complex I inhibitor, and 1 µM antimycin A, a complex III inhibitor. PC9 and PC9M2 cells were plated at a density of 35,000 cells a day before the experiment. Cells were washed with unbuffered assay medium supplemented with 25 mM glucose, 1 mM pyruvate, and 4 mM glutamine (same as in the culture medium) and incubated for 1 h at 37 °C without CO_2_ in unbuffered assay medium before the experiment. The PC9M2 cells proliferated 1.2-times faster than the PC9 cells at the time of measurement, which was 14 h after seeding the cells. Thus, we normalized OCR by cell number.

#### Xenograft assay

1 × 10^6^ cells were suspended in 50 µl RPMI-1640, mixed with 50 µl of Matrigel (354234, Corning) and injected subcutaneously into 8 weeks old female KSN/Slc mice (SLC). All mice were randomized based on the body weight and allocated into the different groups. Administration of drugs was started when tumor size exceeded 100–150 mm^2^.

Gefitinib (G0546, Tokyo Chemical Industry) dissolved in DMSO was diluted in a 30% polyethylene glycol 300, 5% Tween80 solution and given every 2 days by oral gavage at a concentration of 50 mg/Kg. MYLS22 was diluted in the same solvent and given peritumorally every 2 days (25 mg/Kg). The control groups were given DMSO. 18 days after the treatment, the mice were euthanized, and their tumors were harvested. During the experiments, the researchers were aware of the group assignments. Mouse experiments were approved by the Kanazawa University Institutional Animal Care and Use Committee (protocol AP-153426).

#### Immunohistochemistry (IHC) and TUNEL staining

For IHC and TUNEL, xenograft tumors were fixed in 4% paraformaldehyde in PBS for 24 h at room temperature and transferred into 70% ethanol. Paraffin sections were stained with hematoxylin and eosin, or immunostained with anti-Ki67 (1:400, Cell Signaling Technology, #D3B5) as follows. The sections were boiled in 10 mM citrate (pH 6.0) buffer for 10 min, treated with peroxidase block (10062747, Dako) for 10 min, washed by running water and PBS, pretreated with PBS containing 5% goat serum, then 0.1% Triton X-100 and 1% BSA at room temperature for 10 min. Sections were incubated with the primary antibodies at 4 °C overnight, and secondary antibodies at room temperature for 1 h. The IHC-positive signals were visualized using DAB kit (K1390, Dako). For detection of apoptotic cells, TUNEL staining was performed using in situ cell death detection kit TMR red (Roche Diagnostics) according to manufacturer’s instructions.

#### Calculation of combination synergy factor

Synergism between gefitinib and MYLS22 was quantified with SynergyFinder supplied as an R package (https://bioconductor.org/packages/release/bioc/html/synergyfinder.html). Synergy score based on the zero-potency interaction model is visualized as a two-dimensional landscape. The overall levels of synergy effects of the two compounds are shown as delta score.

#### Statistical analysis

Data are shown as mean ± SEM values of the indicated number of independent experiments. Data from individual experiments are plotted as dots except in the OCR experiments which represent mean ± SEM of the indicated number of independent experiments. OriginPro 9.1 was used for statistical analysis. The sample size was predetermined based on published literature and previous lab experience. No statistical methods were used to predetermine the sample size. Normal distribution of data was verified by a Shapiro–Wilkinson test. The homogeneity of the variance was calculated using Levene’s test. In cases where data fulfill specific criteria for normality and homoscedasticity, appropriate parametric tests were utilized to evaluate significance; conversely, when data exhibited a non-normal and unequal distribution, relevant non-parametric tests were applied, as noted in the Figure legends. Sample size and *P* values are indicated in the figure legends, and *P* < 0.05 was considered significant.

## Supplementary information

Supplementary material

Original Data File

Reproducibility checklist

## Data Availability

Uncropped versions of the Western blots are available as original data files. The other datasets generated during and/or analyzed during the current study are available from the corresponding authors upon reasonable request.
