# Supplementary material for: Inhibition of the mitochondria-shaping protein Opa1 restores sensitivity to Gefitinib in a lung adenocarcinomaresistant cell line
Source: Cell Death Dis. 2023 Apr 5;14(4):241. doi: 10.1038/s41419-023-05768-2 (PMC10076284; doi:10.1038/s41419-023-05768-2)
Supplement: Supplementary file 1 — Supplementary material [file 41419_2023_5768_MOESM1_ESM.docx]

**Inhibition of the mitochondria-shaping protein Opa1 restores sensitivity to Gefitinib in a lung adenocarcinoma resistant cell.**

Masafumi Noguchi et al

**Supplementary online material**

**Supplementary Figures**

**
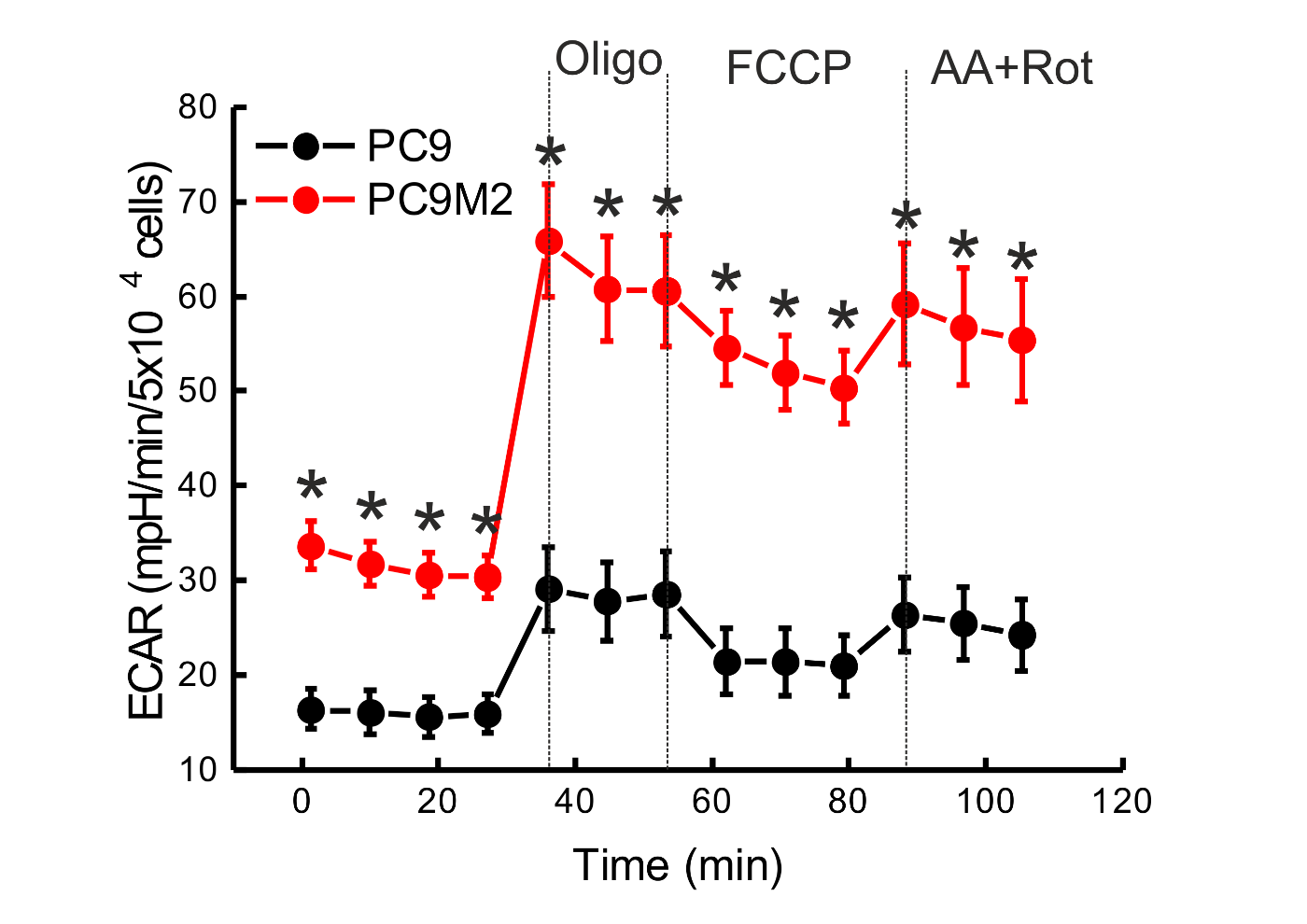
**

**Supplementary Figure 1.** **PC9M2 cells display higher extracellular acidification rates.**

Extracellular acidification rates were measured by Seahorse. Where indicated, 0.75µM Oligomycin (Oligo), 1µM FCCP, 1µM Antimycin A (AA) and 1µM Rotenone (Rot) were injected. Data represent mean ± SEM of 3 independent experiments. p-values were calculated using a two-sided Student’s t-test (*, p<0.05).


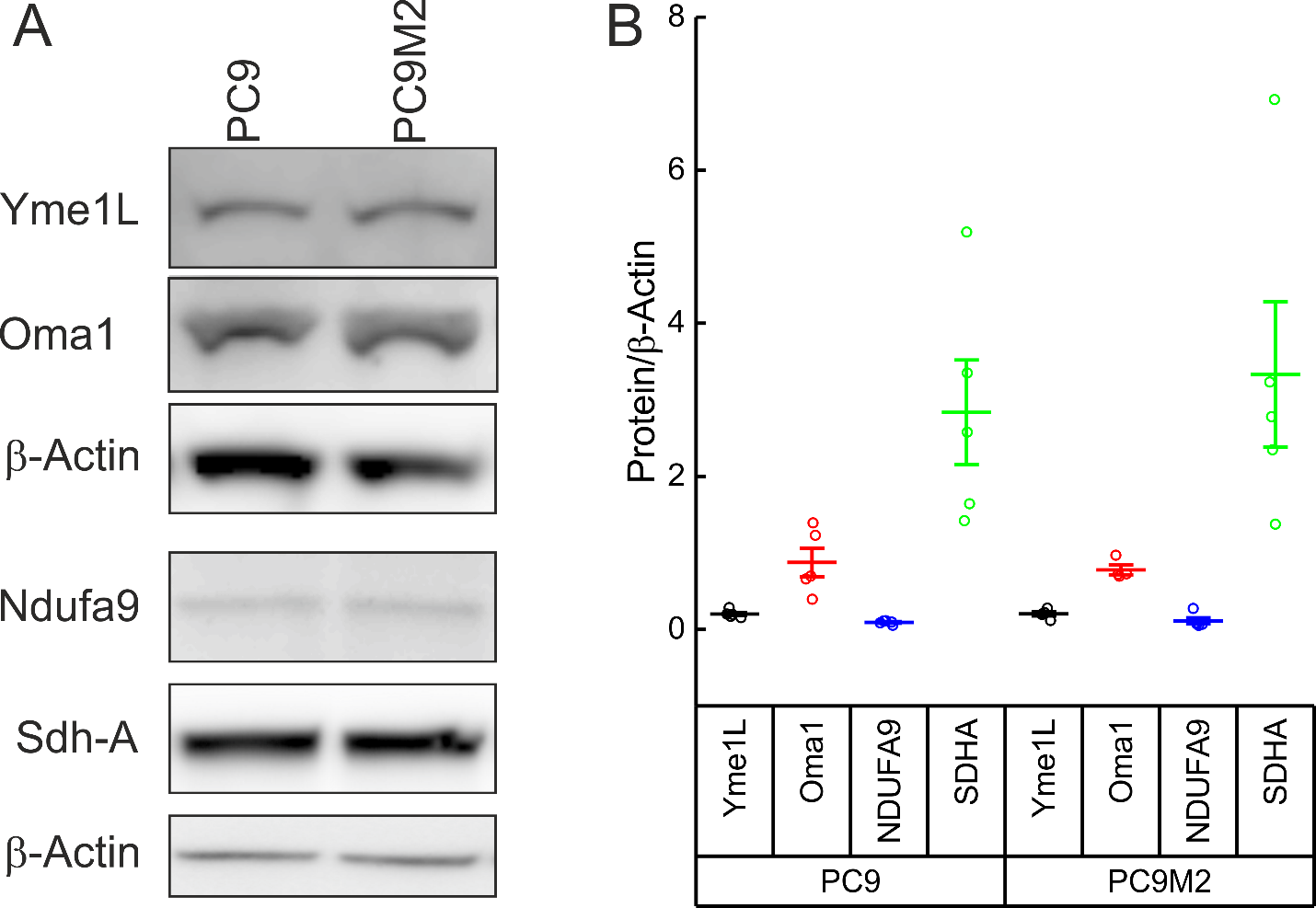


**Supplementary Figure 2. Levels of mitochondrial proteins are similar in PC9 and PC9M2 cells.**

**A** PC9 and PC9M2 cells were lysed, and equal amounts of proteins (40 µg) were separated by SDS-PAGE and immunoblotted using the indicated antibodies.

**B** Mean± SEM of densitometric data from 5 independent experiments in **A**.

**

**

**Supplementary Figure 3. MYLS22 does not sensitize PC9 cells to Gefitinib.**

PC9 cells were treated with 1 µM gefitinib in the presence of the indicated concentrations of MYLS22 and viability was assessed by Annexin-V/PI staining. Data represent mean ± SEM of 5 independent experiments. p-values were calculated using ANOVA with Tukey's multiple comparison test.

**
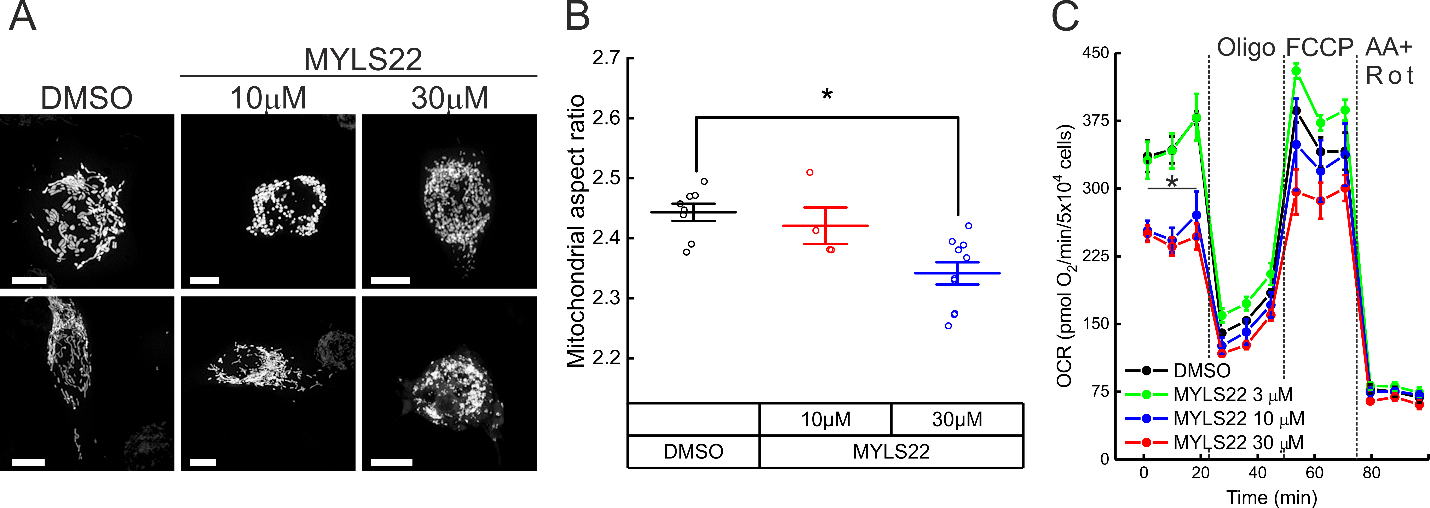
**

**Supplementary Figure 4. MYLS22 causes mitochondrial fragmentation and reduces OCR in PC9M2 cells.**

**A** Representative maximum projections of confocal Z-stacks of mtYFP fluorescence in PC9M2 cells transfected with mtYFP. Twenty-four hours after transfection, PC9M2 cells were treated with the indicated concentrations of the indicated compounds for 48 h and confocal Z-stacks were acquired. Bar 10 μm.

**B** Mitochondrial aspect ratio in PC9M2 cells in experiments as in **A**. Data represent mean ± SEM of 3 independent experiments. p-values were calculated using a non-parametric Scheffe test (*, p<0.05).

**C** Oxygen consumption rates were measured by Seahorse. Where indicated, 0.75 µM Oligomycin (Oligo), 1 µM FCCP, 1 µM Antimycin A (AA) and 1 µM Rotenone (Rot) were injected. Data represent mean ± SEM of 4 independent experiments. p-values were calculated using a non-parametric Scheffe test (*, p<0.05).
