# Supplementary figures and images for: Inhibition of the mitochondria-shaping protein Opa1 restores sensitivity to Gefitinib in a lung adenocarcinomaresistant cell line
Source: Cell Death Dis. 2023 Apr 5;14(4):241. doi: 10.1038/s41419-023-05768-2 (PMC10076284; doi:10.1038/s41419-023-05768-2)

Fig1G

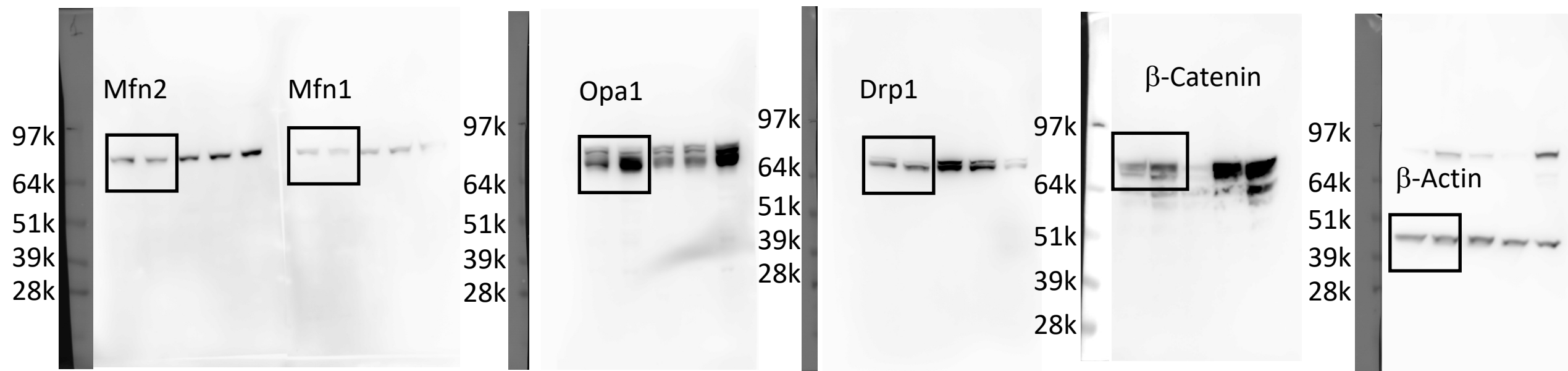

Fig2A

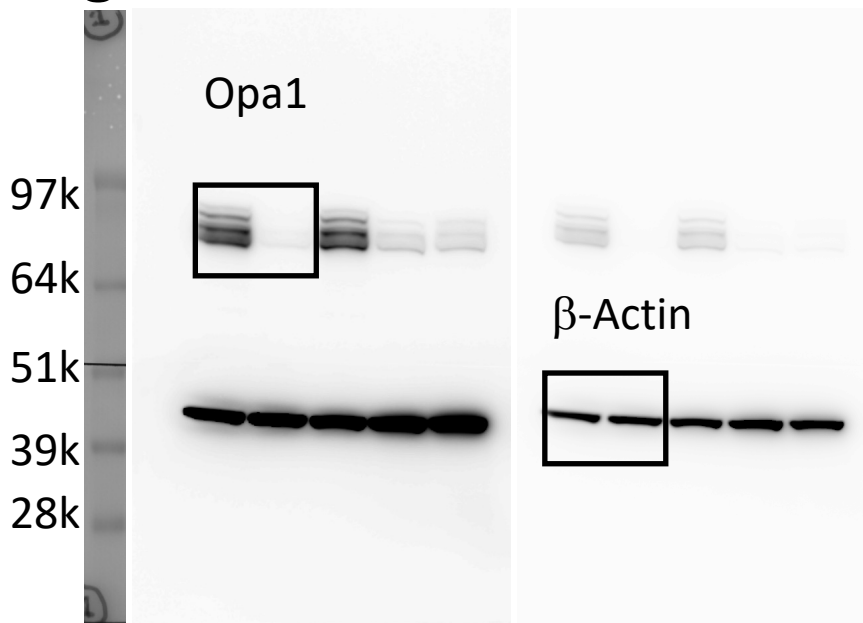

Fig supp 2A

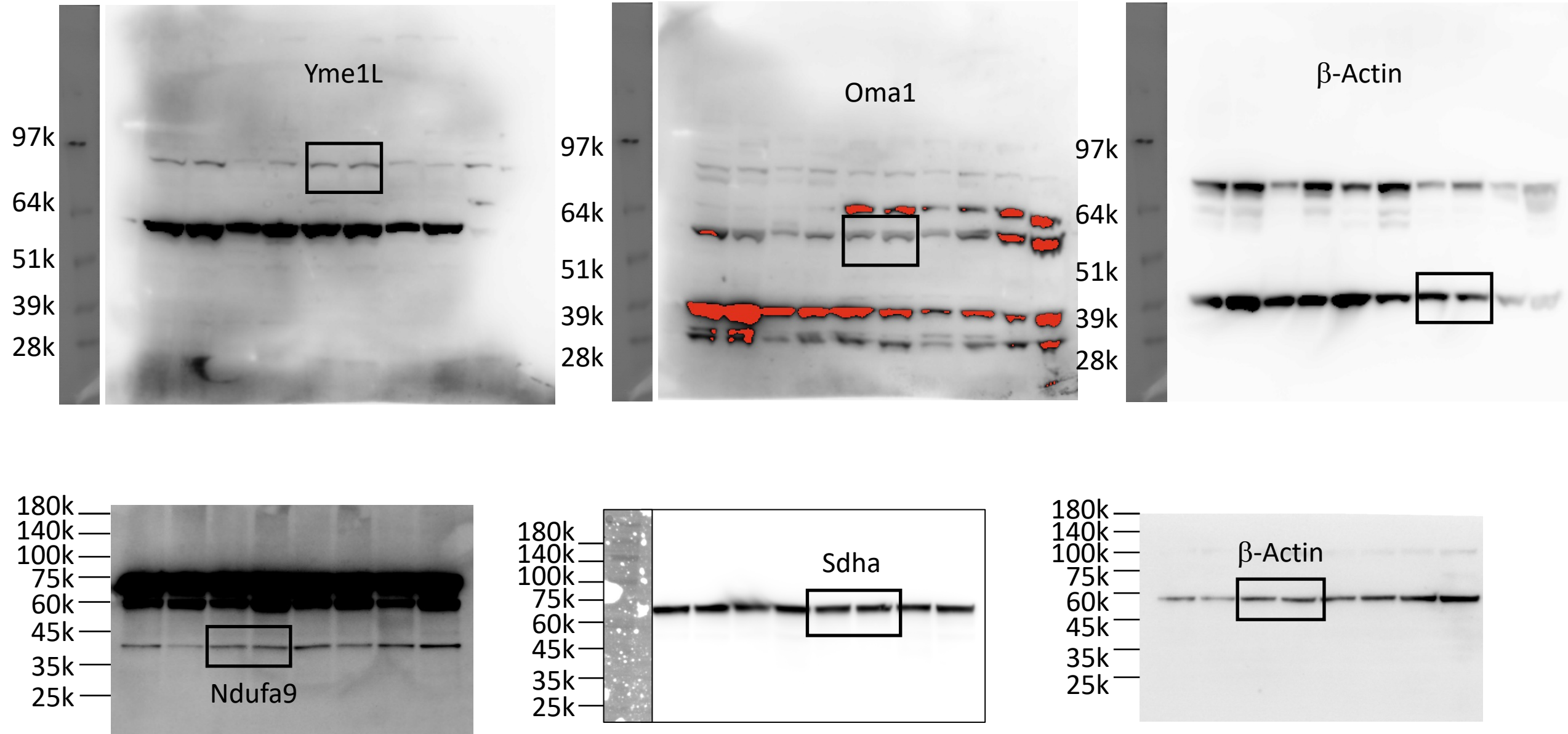

Supplement: Supplementary file 2 — Original Data File [file 41419_2023_5768_MOESM2_ESM.pdf]
